# Hepatitis C Virus Testing in Adults Living with HIV: A Need for Improved Screening Efforts

**DOI:** 10.1371/journal.pone.0102766

**Published:** 2014-07-17

**Authors:** Baligh R. Yehia, Ramin S. Herati, John A. Fleishman, Joel E. Gallant, Allison L. Agwu, Stephen A. Berry, P. Todd Korthuis, Richard D. Moore, Joshua P. Metlay, Kelly A. Gebo, for the HIV Research Network

**Affiliations:** 1 Department of Medicine, University of Pennsylvania Perelman School of Medicine, Philadelphia, Pennsylvania, United States of America; 2 Leonard Davis Institute of Health Economics, University of Pennsylvania, Philadelphia, Pennsylvania, United States of America; 3 Center for Financing, Access, and Cost Trends, Agency for Healthcare Research and Quality, Rockville, Maryland, United States of America; 4 Southwest Care Center, Santa Fe, New Mexico, United States of America; 5 Department of Medicine, Johns Hopkins University School of Medicine, Baltimore, Maryland, United States of America; 6 Department of Medicine, Oregon Health and Sciences University, Portland, Oregon, United States of America; 7 General Medicine Division, Massachusetts General Hospital, Boston, Massachusetts, United States of America; Temple University School of Medicine, United States of America

## Abstract

**Objectives:**

Guidelines recommend hepatitis C virus (HCV) screening for all people living with HIV (PLWH). Understanding HCV testing practices may improve compliance with guidelines and can help identify areas for future intervention.

**Methods:**

We evaluated HCV screening and unnecessary repeat HCV testing in 8,590 PLWH initiating care at 12 U.S. HIV clinics between 2006 and 2010, with follow-up through 2011. Multivariable logistic regression examined the association between patient factors and the outcomes: HCV screening (≥1 HCV antibody tests during the study period) and unnecessary repeat HCV testing (≥1 HCV antibody tests in patients with a prior positive test result).

**Results:**

Overall, 82% of patients were screened for HCV, 18% of those screened were HCV antibody-positive, and 40% of HCV antibody-positive patients had unnecessary repeat HCV testing. The likelihood of being screened for HCV increased as the number of outpatient visits rose (adjusted odds ratio 1.02, 95% confidence interval 1.01–1.03). Compared to men who have sex with men (MSM), patients with injection drug use (IDU) were less likely to be screened for HCV (0.63, 0.52–0.78); while individuals with Medicaid were more likely to be screened than those with private insurance (1.30, 1.04–1.62). Patients with heterosexual (1.78, 1.20–2.65) and IDU (1.58, 1.06–2.34) risk compared to MSM, and those with higher numbers of outpatient (1.03, 1.01–1.04) and inpatient (1.09, 1.01–1.19) visits were at greatest risk of unnecessary HCV testing.

**Conclusions:**

Additional efforts to improve compliance with HCV testing guidelines are needed. Leveraging health information technology may increase HCV screening and reduce unnecessary testing.

## Introduction

Up to 25% of people living with HIV (PLWH) in the United States (U.S.) are coinfected with hepatitis C virus (HCV). [1] HIV/HCV coinfection accelerates progression to liver fibrosis, end-stage liver disease, and death compared to HCV monoinfection. [2] Consequently, guidelines recommend that all PLWH be screened for HCV infection upon initiation of HIV care [3]–[6].

Despite these recommendations, HCV screening rates have varied for PLWH. [7]–[10] Among a random sample of 1,329 HIV-infected men who have sex with men (MSM) in care at 8 U.S. HIV clinics between 2004 and 2007, only 54% were ever tested for HCV. [7] Conversely, the U.S. Veterans Health Administration (VA) reported that 96% of the 23,463 HIV-infected veterans in care in 2008 received HCV screening [9].

Screening for HCV is performed by testing for HCV antibody; positive results should be followed by measurement of HCV RNA to differentiate chronic infection from resolved infection. [11] HCV antibody testing in individuals with a prior positive result is unnecessary, as the antibody test remains reactive regardless of whether the infection has cleared. [11] However, PLWH may be at increased risk of unnecessary HCV testing due to the high prevalence of HCV infection and their more frequent use of medical services compared to the general population [3].

Understanding HCV testing practices is important to improving compliance with national guidelines and can help identify areas for future intervention. The goal of this study was to estimate the proportion of PLWH screened for HCV and to identify groups at risk of not being screened. We also assessed unnecessary repeat HCV testing among patients with a prior positive HCV antibody test.

### Methods

The HIV Research Network (HIVRN) is a consortium of clinics that provide care to people living with HIV. [12], [13] Data from 12 sites treating adults, located in the Northeastern (6), Midwestern (1), Southern (2), and Western (3) U.S., were included in this analysis. All patients establishing care at these sites are offered enrollment in the HIVRN; 99% of patients consent to participate. Sites abstract data from medical records and send them to a data-coordinating center after removing personal identifying information. Following quality control and verification, data are combined across sites to produce a uniform database. Institutional review boards (IRBs) at each site (complete list of sites can be found in the acknowledgments) and the data coordinating center at Johns Hopkins University approved the collection and analyses of these data. IRBs at some clinics required written informed consents, while others waived the requirement because only existing anonymized and de-identified data were collected.

HIV-infected adults (age ≥18 years) new to care at HIVRN sites and seen at least twice between January 1, 2006 and December 31, 2010 were included and followed through December 31, 2011. The two-visit criterion was employed to capture patients establishing regular care. To exclude those who may have received care previously, we excluded patients with first recorded HIV RNA ≤400 copies/mL (n = 2,665) and individuals with a history of outpatient HIV visits or use of antiretroviral therapy (ART) prior to HIVRN enrollment (n = 916).

### Variables

Demographic and clinical characteristics (age, sex, race/ethnicity, HIV transmission risk, insurance coverage, CD4 count) at the time of the first outpatient visit were collected for each patient. Patients’ age was divided into 4 groups: 18–29, 30–39, 40–49, and over 50 years old. Race/ethnicity was categorized as non-Hispanic white, non-Hispanic black, Hispanic, and other/unknown. Self-reported HIV transmission behavior categories were MSM, heterosexual transmission (HET), injection drug use (IDU), and other/unknown. Patients who had IDU in combination with another risk factor (e.g. MSM, heterosexual transmission) were classified as IDU. Insurance was categorized as private, Medicaid, Medicare, uninsured, or other/unknown. Patients whose care was funded by Ryan White, those recorded as self-pay, and those covered by local governmental programs were considered to be uninsured. CD4 cell count was categorized as ≤350, 351–500, >500 cells/mm^3^, or missing.

The numbers of outpatient, inpatient, and emergency department visits over the entire study period were collected for each patient. Outpatient visits refer only to primary HIV care appointments made to an HIVRN clinic, and do not include nursing or pharmacy visits, consultations, or other types of appointments.

Dichotomous outcomes were (1) performance of any HCV screening and (2) unnecessary repeat HCV testing, during the observation period. We defined the observation period as the time from date of enrollment to date of last outpatient visit, transfer of care, or death, whichever occurred first. Patients were considered screened for HCV if they had one or more HCV antibody test(s) during the observation period. Unnecessary repeat HCV testing was defined as one or more HCV antibody tests in patients with a prior positive test result.

### Statistical Analysis

We compared demographic and clinical differences in the proportion of patients screened for HCV, HCV antibody positive, and unnecessarily tested for HCV using χ^2^ tests. Multivariable logistic regression was used to estimate the association between patient factors and the two outcomes. Analyses of unnecessary repeat HCV testing were restricted to patients with a prior positive HCV test, for whom unnecessary testing could be measured. In secondary analyses, multivariable logistic regression was used to estimate the association between patient factors and repeat HCV testing (>1 HCV antibody tests) among HCV antibody negative patients. All regression models included indicators for each HIVRN site and length of observation period. Analyses were conducted in STATA 12.1 (College Station, TX).

## Results

**Table 1** presents descriptive information for the 8,590 patients new to care at the 12 HIVRN sites in 2006–2010. Median age at presentation was 39 years, with 16% aged 50 years or older. The sample comprised high proportions of patients who were male (75%), of minority race/ethnicity (71%), and who had either no health insurance or Medicaid at the time of their first outpatient visit (66%). The most common HIV transmission behavior was MSM (46%), followed by HET (37%) and IDU (11%). Excluding patients with missing data (N = 607), the mean first CD4 cell count was 318 cells/mm^3^.

**Table 1:** Demographic and Clinical Characteristics of HIV-infected Patients.

Characteristics	HIV-Infected Patients N = 8,590 (%)
**Age (years)**	
18–29	2,134 (24.84)
30–39	2,355 (27.42)
40–49	2,743 (31.93)
≥50	1,358 (15.81)
**Sex**	
Male	6,481 (75.45)
Female	2,109 (24.55)
**Race/Ethnicity**	
White	2,202 (25.63)
Black	4,142 (48.22)
Hispanic	1,926 (22.42)
Other/Unknown	320 (3.73)
**HIV Risk Factor**	
MSM	3,913 (45.55)
HET	3,180 (37.02)
IDU	977 (11.37)
Other/Unknown	520 (6.05)
**Insurance**	
Private	1,270 (14.78)
Medicaid	2,334 (27.17)
Medicare	630 (7.33)
Ryan White/Uninsured	3,334 (38.81)
Other/Unknown	1,022 (11.90)
**First CD4 Cell Count (cell/mm^3^)**	
≤350	4,795 (55.82)
351–500	1,501 (17.47)
>500	1,687 (19.64)
Missing	607 (7.07)
**Number of Outpatient HIV Visits per Year, mean (SD)** *	5.03 (3.12)
**Number of Inpatient Visits per Year, mean (SD)** *	0.34 (0.93)
**Number of Emergency Department Visits per Year, mean (SD)** *	0.60 (1.61)
**Observation Time (years)**	
1	2,338 (27.22)
2	2,010 (23.40)
3	1,486 (17.30)
4	1,220 (14.20)
5	968 (11.27)
6	569 (6.61)

**Abbreviations:** HET, heterosexual transmission; IDU, injection drug use; MSM, men who have sex with men.

*Mean number of outpatient, inpatient, and emergency department visits over the observation period were: 13.92 (standard deviation12.03), 0.75 (1.86), and 1.41 (3.70), respectively.

**Figure 1:**
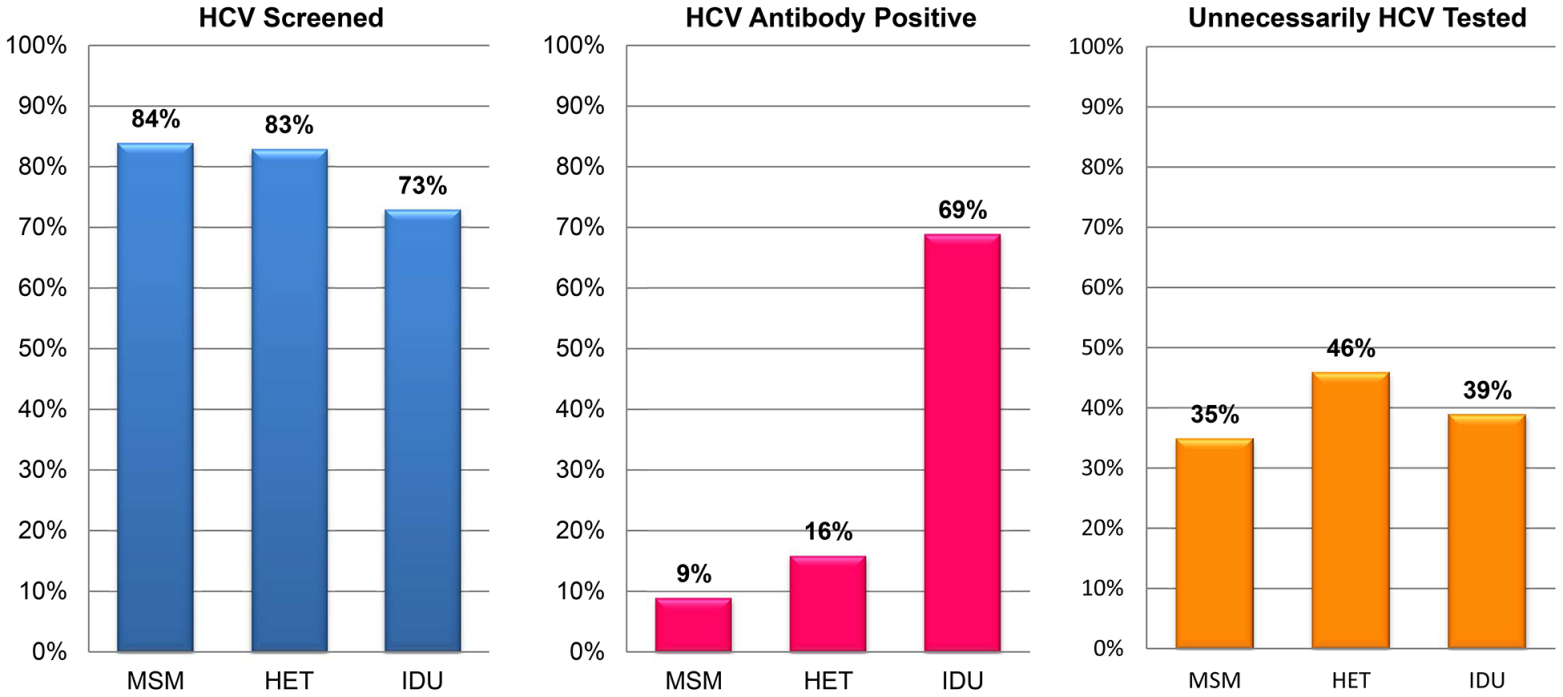
Proportion of Patients Screened for HCV Infection, HCV Antibody Positive, and Unnecessarily Tested for HCV Infection by HIV Transmission Behavior. Abbreviations: HET, heterosexual transmission; IDU, injection drug use; MSM, men who have sex with men.

**Figure 2:**
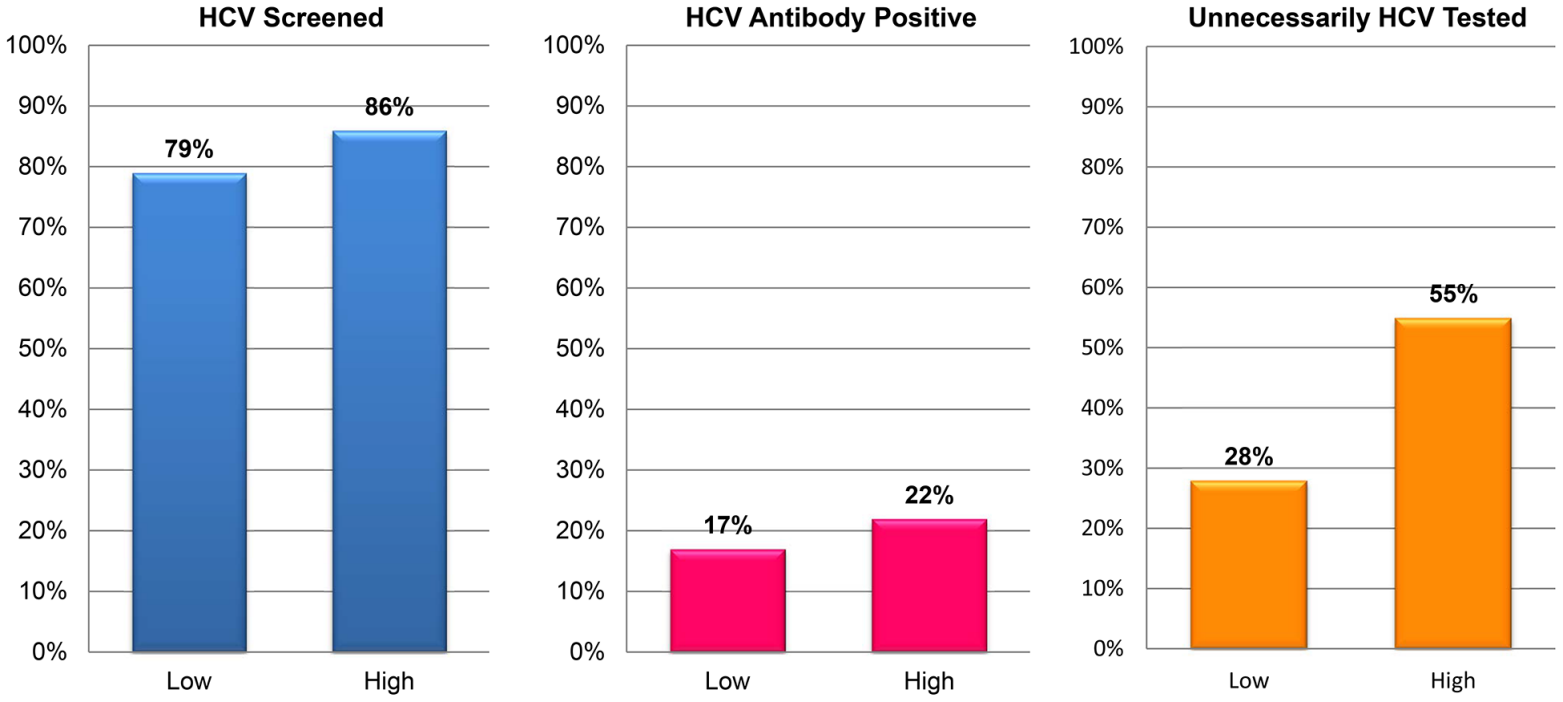
Proportion of Patients Screened for HCV Infection, HCV Antibody Positive, and Unnecessarily Tested for HCV Infection by HIV Outpatient Utilization. Note: The mean value divided the number of outpatient HIV visits during the observation period (13.92) into two groups – low and high.

In total, 7,023 patients (82% of 8,590) were screened for HCV. (**Table 2**) Among those screened, 92% were tested within 1 year of entering care, 96% within 2 years, and 98% within 3 years. HCV screening rates varied *(P*<0.05) by HIV transmission behavior: 84% for MSM, 83% for heterosexual (HET), and 73% for injection drug use (IDU). **(** **Figure 1** **)** Individuals with Medicaid (84%) or Ryan White/uninsured (85%) were more likely to be tested (*P*<0.05) than those with private insurance (79%) and Medicare (80%). Using the mean number of outpatient visits during the study period as a divider, 79% of patients with ≤14 visits were screened compared to 86% of patients who attended more than 14 visits (*P*<0.05). **(** **Figure 2** **)**.

**Table 2:** Proportion of Patients Screened for HCV Infection, HCV Antibody Positive, and Unnecessarily Tested for HCV Infection (2006–2011).

Characteristics	Screened N = 7,023 (%)	Antibody Positive N = 1,283 (%)	Unnecessarily Tested N = 510 (%)
**Age (years)** ‡			
18–29	1,766 (82.76)	93 (5.27)	33 (35.48)
30–39	1,946 (82.63)	248 (12.74)	97 (39.11)
40–49	2,229 (81.26)	562 (25.21)	232 (41.28)
≥50	1,082 (79.68)	380 (35.12)	148 (38.95)
**Sex** ‡			
Male	5,304 (81.84)	933 (17.59)	369 (39.55)
Female	1,719 (81.51)	350 (20.36)	141 (40.29)
**Race/Ethnicity**			
White	1,804 (81.93)	336 (18.63)	119 (35.42)
Black	3,373 (81.43)	627 (18.59)	264 (42.11)
Hispanic	1,578 (81.93)	284 (18.00)	116 (40.85)
Other/Unknown	268 (83.75)	36 (13.43)	11 (30.56)
**HIV Risk Factor** † ‡ §			
MSM	3,273 (83.64)	295 (9.01)	104 (35.25)
HET	2,630 (82.70)	424 (16.12)	195 (45.99)
IDU	714 (73.08)	493 (69.05)	194 (39.35)
Other/Unknown	406 (78.08)	71 (17.49)	17 (23.94)
**Insurance** † ‡			
Private	999 (78.66)	82 (8.21)	26 (31.71)
Medicaid	1,963 (84.10)	534 (27.20)	214 (40.07)
Medicare	508 (80.63)	119 (23.43)	47 (39.50)
Ryan White/Uninsured	2,835 (85.03)	408 (14.39)	171 (41.91)
Other/Unknown	718 (70.25)	140 (19.27)	52 (37.14)
**First CD4 Cell Count (cell/mm^3^)** † ‡			
≤350	4,038 (84.21)	778 (19.27)	318 (40.87)
351–500	1,252 (83.41)	192 (15.34)	81 (42.19)
>500	1,388 (82.28)	247 (17.80)	87 (35.22)
Missing	345 (56.84)	66 (19.13)	24 (36.36)
**Number of Outpatient HIV Visits** * † ‡ §			
≤14	4,153 (79.21)	806 (16.64)	201 (27.72)
>14	2,870 (85.75)	477 (21.90)	309 (55.38)
**Number of Inpatient Visits** * ‡ §			
0	4,845 (81.35)	725 (17.46)	290 (35.98)
≥1	2,178 (82.69)	558 (19.44)	220 (46.12)
**Number of Emergency Department Visits** * §			
0	4,434 (81.34)	785 (17.70)	286 (36.43)
≥1	2,589 (82.48)	498 (19.24)	224 (44.98)
**Observation Time (years)** † ‡ §			
1	1,812 (77.50)	368 (20.31)	75 (20.38)
2	1,617 (80.45)	226 (13.98)	75 (33.98)
3	1,261 (84.86)	225 (17.84)	98 (43.56)
4	1,062 (87.05)	200 (18.83)	102 (51.00)
5	802 (82.85)	152 (18.95)	93 (63.18)
6	469 (82.57)	112 (23.88)	67 (59.82)

**Abbreviations:** HET, heterosexual transmission; IDU, injection drug use; MSM, men who have sex with men.

*Continuous variables were dichotomized to facilitate calculation of proportions. The mean value divided the number of outpatient HIV visits during the observation period into two groups; whereas the number of inpatient and emergency department visits during the observation period differentiated between 0 and 1 or more visits.

† *P*<0.05 when comparing differences in the proportion screened for HCV using the χ^2^ test.

‡ *P*<0.05 when comparing differences in the proportion HCV antibody positive using the χ^2^ test.

§ *P*<0.05 when comparing differences in the proportion unnecessary tested for HCV using the χ^2^ test.

In multivariate analyses, the likelihood of being screened for HCV increased as the number of outpatient visits rose (adjusted odds ratio (AOR) = 1.02; 95% confidence interval (CI) = 1.01–1.03). Compared to MSM, patients with IDU risk were less likely to be screened (AOR = 0.63, 95% CI 0.52–0.78). Individuals with Medicaid were more likely to be screened than those with private insurance (AOR = 1.30, 95% CI = 1.04–1.62). (**Table 3**).

**Table 3:** Factors Associated with HCV Screening and Unnecessary Repeat HCV Testing.

Characteristics	HCV Screening	Unnecessary Testing
	Adjusted Odds Ratio (95% CI)	Adjusted Odds Ratio (95% CI)
**Age (years)**		
18–29	1.00 (reference)	1.00 (reference)
30–39	1.04 (0.87–1.24)	1.33 (0.73–2.42)
40–49	0.91 (0.77–1.09)	1.26 (0.72–2.21)
≥50	0.88 (0.72–1.08)	1.00 (0.56–1.83)
**Sex**		
Male	1.00 (reference)	1.00 (reference)
Female	1.00 (0.85–1.18)	0.92 (0.68–1.25)
**Race/Ethnicity**		
White	1.00 (reference)	1.00 (reference)
Black	1.01 (0.84–1.20)	1.13 (0.79–1.62)
Hispanic	0.85 (0.70–1.04)	0.98 (0.65–1.47)
Other/Unknown	1.36 (0.94–1.97)	1.64 (0.57–4.73)
**HIV Risk Factor**		
MSM	1.00 (reference)	1.00 (reference)
HET	0.96 (0.81–1.14)	1.78 (1.20–2.65)
IDU	0.63 (0.52–0.78)	1.58 (1.06–2.34)
Other/Unknown	1.08 (0.81–1.43)	1.34 (0.63–2.82)
**Insurance**		
Private	1.00 (reference)	1.00 (reference)
Medicaid	1.30 (1.04–1.62)	1.17 (0.64–2.14)
Medicare	1.01 (0.76–1.33)	1.13 (0.56–2.27)
Ryan White/Uninsured	1.21 (0.97–1.50)	1.23 (0.66–2.30)
Other/Unknown	0.93 (0.70–1.23)	0.74 (0.35–1.54)
**CD4 Cell Count (cell/mm^3^)**		
≤350	1.00 (reference)	1.00 (reference)
351–500	0.98 (0.83–1.17)	1.18 (0.81–1.71)
>500	0.90 (0.76–1.06)	0.82 (0.56–1.17)
Missing	0.22 (0.17–0.29)	0.65 (0.33–1.29)
**Number of Outpatient HIV Visits**	1.02 (1.01–1.03)	1.03 (1.01–1.04)
**Number of Inpatient Visits**	0.99 (0.95–1.03)	1.09 (1.01–1.19)
**Number of Emergency Department Visits**	0.99 (0.97–1.01)	0.96 (0.91–1.01)
**Observation Time (years)**		
1	1.00 (reference)	1.00 (reference)
2	1.26 (1.07–1.50)	1.80 (1.17–2.76)
3	1.70 (1.39–2.10)	2.68 (1.73–4.16)
4	2.14 (1.67–2.73)	3.64 (2.25–5.88)
5	1.73 (1.31–2.29)	4.18 (2.37–7.39)
6	1.20 (0.86–1.67)	3.96 (2.17–7.25)

**Abbreviations:** CI, confidence interval; HET, heterosexual transmission; IDU, injection drug use; MSM, men who have sex with men.

Among patients screened for HCV, 1,283 (18% of 7,023) had a positive test result. (**Table 2**) The percentage of patients identified as HCV antibody positive varied (*P*<0.05) by age, sex, and HIV transmission behavior. Persons with public insurance and greater numbers of outpatient and inpatient visits had higher proportions of HCV antibody positivity than those with private insurance and lower numbers of outpatient and inpatient visits, respectively (*P*<0.05).

Of patients with a positive HCV antibody, 510 (40% of 1,283) had an unnecessary repeat HCV test. (**Table 2**) Sixty-five percent had one unnecessary test, 19% had two, 10%, had three, and 6% had four or more unnecessary tests. Unnecessary testing varied by HIV risk behavior, with 35% of MSM, 46% of HET, and 39% of IDU undergoing repeat testing (*P*<0.05). **(** **Figure 1** **)** Individuals with greater numbers of outpatient, inpatient, and emergency department visits had higher proportions of unnecessary testing than those with lower numbers of outpatient, inpatient, and emergency department visits, respectively (*P*<0.05) **(** **Figure 2** **)**.

In multivariate logistic regression analysis, persons with HET (AOR = 1.78, 95% CI = 1.20–2.65) and IDU (AOR = 1.58, 95% CI = 1.06–2.34) risk and those with higher numbers of outpatient (AOR = 1.03, 95% CI = 1.01–1.04) and inpatient (AOR = 1.09, 95% CI = 1.01–1.19) visits were more likely to be tested unnecessarily (**Table 3**).

We identified 5,740 patients who were HCV antibody negative throughout the observation period. Of these, 2,447 (43% of 5,740) had a repeat HCV test. Fifty-five percent had one repeat test, 24% had two, 11%, had three, and 10% had four or more repeat tests. In multivariate analyses, the likelihood of having a repeat HCV test increased as the number of outpatient visits (AOR = 1.03, 95% CI = 1.02–1.04) and inpatient visits (AOR = 1.14, 95% CI = 1.08–1.20) increased. Compared to MSM, patients with IDU risk were more likely to have repeat testing (AOR = 1.54, 95% CI = 1.04–2.29). Similarly, individuals with Medicaid were more likely to have repeat testing than those with private insurance (AOR = 1.54, 95% CI = 1.10–2.17). **(Appendix S1)**.

## Discussion

Despite recommendations that all HIV-infected patients be screened for HCV infection upon initiation of HIV care, [3]–[6] only 82% of patients were screened. This screening rate is higher than the 16–54% found in some prior studies but is lower than the VA (96%). [7]–[10] The VA has prioritized HCV screening, [14] as veterans have a higher prevalence of HCV infection compared to the general population. [15] The VA also uses an integrated electronic medical record system that is easily accessible by practitioners at multiple sites and generates preventive care and screening reminders. This combination of prioritized HCV screening and sophisticated health information technology may serve as a model for other HIV clinics. In addition, alerting patients and providers when screenings are due, evaluating provider performance and offering feedback, delivering one-on-one and group education to patients, and using patient and provider incentives have been identified as additional tools for improving compliance with screening guidelines [16]–[20].

Similar to unnecessary HCV testing rates reported among non-HIV infected individuals, [21] 40% of HIV-infected HCV antibody positive patients in this study had unnecessary duplicate HCV tests. Multiple factors could contribute to the high number of inappropriate tests, including failure to check previous test results, lack of awareness that a test has already been performed, and inadequate distribution of patient information across multiple medical record systems. [22], [23] In a survey of 283 primary care physicians and residents, 17–32% reported having no reliable method to track test results, suggesting the need to develop effective and user-friendly methods for monitoring test results [23].

Computer provider order entry (CPOE) with embedded decision-support tools may provide one solution for reducing repetitive testing. [24], [25] In one study, point-of-care prompts decreased redundant testing by 24%, demonstrating the feasibility of CPOE decision-support tools to reduce unnecessary testing. [24] However, given the widespread adoption of electronic health records and the U.S. government’s emphasis on health information technology, many practices may already have access to these resources [26], [27].

Injection drug users are at increased risk of HCV and disproportionally account for 80% of new infections and 60% of existing cases in developed countries. [28] Despite this risk, in our cohort, injection drug users were less likely to be screened for HCV than MSM. This finding may be due, in part, to the multiple comorbidities and psychosocial barriers facing many people with IDU. Providers are challenged with managing drug addiction; treating co-occurring conditions, including HIV infection and mental illness; and addressing competing needs (e.g. lack of housing, insurance, social support) during a 20–30 minute office visit. [29]–[33] Deferral of preventative care services, as typically occurs during complicated visits, and the need to review lengthy medical records to verify prior test results may lead to lower HCV screening. Conversely, the recent emergence of sexually transmitted acute HCV infection may influence HCV testing in MSM. [34] Increased awareness of HCV transmission in this group by both patients and providers may have contributed to the higher screening rate [35].

In our study, greater use of health care services was significantly associated with increased HCV testing, both appropriate and unnecessary. Prior research demonstrates that the number of laboratory tests performed rises with the number of health system interactions. [29] In addition, higher health care utilization can compromise care coordination, which may lead to over treatment and testing. [36]–[39] Accordingly, improving care coordination and addressing the needs of high users of health care services have been identified as key targets for reducing waste in the U.S. health care system [36], [40]–[42].

The current analysis has several limitations. First, sites in our sample are not nationally representative, although they do encompass a broad geographic distribution and a variety of demographic and clinical characteristics of patients, thereby improving generalizeability compared to single-site studies. Second, our data only capture laboratory tests performed at or reported to the site of care. It is possible that patients may be tested for HCV during consultation with other physicians or during hospitalization at outside institutions. Third, some patients may have been tested for HCV prior to entering HIV care. If the results of these test(s) are accurately documented and reported, some providers may not pursue HCV testing. Lastly, our analyses did not focus on the cost of unnecessary HCV testing. Additional studies are needed to estimate how repeat HCV testing impacts health expenditures. These studies should not only consider the added cost of HCV antibody testing but also potential downstream costs (e.g. HCV viral load testing, genotype testing, radiologic studies, and provider referrals).

In summary, 82% of HIV-infected patients were screened for HCV, 18% of those screened were HCV antibody-positive, and 40% of HCV antibody-positive patients had unnecessary repeat HCV testing. Additional efforts are needed to increase HCV screening, particularly for PLWH with IDU risk. In low prevalence populations, serum-pooling strategies may represent cost-effective techniques to improve screening. [43], [44] Leveraging health information technology may be useful for both increasing screening and reducing unnecessary testing.

## Supporting Information

Appendix S1 Proportion of HCV Antibody Negative Patients with Repeat HCV Testing and Factors Associated with Repeat HCV Testing.
(DOC)

## References

[1] Kim AY, Onofrey S, Church DR (2007) An epidemiologic update on hepatitis C infection in persons living with or at risk of HIV infection. J Infect Dis Suppl 1: S1–6. 10.1093/infdis/jis927 PMC3565593 23390299

[2] Operskalski EA, Kovacs A (2011) HIV/HCV co-infection: pathogenesis, clinical complications, treatment, and new therapeutic technologies. Curr HIV/AIDS Rep 8(1): 12–22. 21221855 10.1007/s11904-010-0071-3 PMC3035774

[3] Kaplan JE, Benson C, Holmes KH, Brooks JT, Pau A, et al (2009) Guidelines for the prevention and treatment of opportunistic infections in HIV-infected adults and adolescents: recommendations from the Centers for Disease Control and Prevention, the National Institutes of Health, and the HIV Medicine Association of the Infectious Diseases Society of America. MMWR Recomm Rep 58: 1–207. 19357635

[4] Aberg JA, Kaplan JE, Libman H, Emmanuel P, Anderson JR, et al (2009) Primary care guidelines for the management of persons infected with human immunodeficiency virus: 2009 update by the HIV Medicine Association of the Infectious Diseases Society of America. Clin Infect Dis 49(5): 651–681. 19640227 10.1086/605292

[5] Centers for Disease Control and Prevention (2012) Recommendations for the Identification of Hepatitis C Virus Infection among Persons Born from 1945 through 1965. Morb Mortal Wkly Rep 61: 1–32. 22895429

[6] Department of Health and Human Services (2013) Guidelines for the Use of Antiretroviral Agents in HIV-1-Infected Adults and Adolescents. Available at: http://www.aidsinfo.nih.gov/contentfiles/lvguidelines/adultandadolescentgl.pdf. Accessed May 15, 2014.

[7] Hoover KW, Butler M, Workowski KA, Follansbee S, Gratzer B, et al (2012) Low rates of hepatitis screening and vaccination of HIV-infected MSM in HIV clinics. Sex Transm Dis 39: 349–353. 22504597 10.1097/OLQ.0b013e318244a923

[8] Jonckheere S, Vincent A, Belkhir L, Wilmes D, Vandercam B, et al (2013) Adherence to Screening Guidelines for Hepatitis C Among HIV-Positive Patients. AIDS Patient Care STDS 27: 317–319. 23682636 10.1089/apc.2013.0096 PMC3671626

[9] Veterans Health Administration (2009) The State of Care for Veterans with HIV/AIDS. 2009. Available at http://www.hiv.va.gov/HIV/provider/state-of-care/index.asp. Accessed May 15, 2014.

[10] Linas BP, Hu H, Barter DM, Horberg M (2014) Hepatitis C screening trends in a large integrated health system. Am J Med 127: 398–405. 24486288 10.1016/j.amjmed.2014.01.012 PMC3999187

[11] Centers for Disease Control and Prevention (2013) Testing for HCV infection: an update of guidance for clinicians and laboratorians. MMWR Morb Mortal Wkly Rep 62: 362–365. 23657112 PMC4605020

[12] Yehia BR, Gebo KA, Hicks PB, Korthuis PT, Moore RD, Ridore M, et al (2008) Structures of care in the clinics of the HIV Research Network. AIDS Patient Care STDS 22: 1007–1013. 19072107 10.1089/apc.2008.0093 PMC2605637

[13] Yehia BR, Agwu AL, Schranz A, Korthuis PT, Gaur AH, Rutstein R, et al (2013) Conformity of pediatric/adolescent HIV clinics to the patient-centered medical home care model. AIDS Patient Care STDS 27: 272–279. 23651104 10.1089/apc.2013.0007 PMC3651683

[14] Veterans Health Administration (2013) Veterans Health Administration Directive 1300.01: National Viral Hepatitis Program. Available at http://www1.va.gov/vhapublications/ViewPublication.asp?pub_ID=1586. Accessed May 15, 2014.

[15] Chak E, Talal AH, Sherman KE, Schiff ER, Saab S (2011) Hepatitis C virus infection in USA: an estimate of true prevalence. Liver Int 31: 1090–1101. 21745274 10.1111/j.1478-3231.2011.02494.x

[16] Baron RC, Melillo S, Rimer BK, Coates RJ, Kerner J, Habarta N, et al (2010) Intervention to increase recommendation and delivery of screening for breast, cervical, and colorectal cancers by healthcare providers a systematic review of provider reminders. Am J Prev Med 38: 110–117. 20117566 10.1016/j.amepre.2009.09.031

[17] Legorreta AP, Hasan MM, Peters AL, Pelletier KR, Leung KM (1997) An intervention for enhancing compliance with screening recommendations for diabetic retinopathy. A bicoastal experience. Diabetes Care 20: 520–523. 9096973 10.2337/diacare.20.4.520

[18] White P, Kenton K (2013) Use of electronic medical record-based tools to improve compliance with cervical cancer screening guidelines: effect of an educational intervention on physicians’ practice patterns. J Low Genit Tract Dis 17: 175–181. 23343700 10.1097/LGT.0b013e3182607137

[19] Asonganyi E, Vaghasia M, Rodrigues C, Phadtare A, Ford A, et al (2013) Factors affecting compliance with clinical practice guidelines for pap smear screening among healthcare providers in africa: systematic review and meta-summary of 2045 individuals. PLoS One 8(9): e72712. 24069156 10.1371/journal.pone.0072712 PMC3771969

[20] Community Preventive Services Task Force (2012) Updated recommendations for client- and provider-oriented interventions to increase breast, cervical, and colorectal cancer screening. Am J Prev Med 43: 92–96. 22704753 10.1016/j.amepre.2012.04.008

[21] Groom H, Dieperink E, Nelson DB, Garrard J, Johnson JR, et al (2008) Outcomes of a Hepatitis C screening program at a large urban VA medical center. J Clin Gastroenterol 42: 97–106. 18097298 10.1097/MCG.0b013e31802dc56f

[22] Kwok J, Jones B (2005) Unnecessary repeat requesting of tests: an audit in a government hospital immunology laboratory. J Clin Pathol. 58: 457–462. 10.1136/jcp.2004.021691 PMC1770647 15858114

[23] Boohaker EA, Ward RE, Uman JE, McCarthy BD (1996) Patient notification and follow-up of abnormal test results. A physician survey. Arch Intern Med 156: 327–331. 8572844

[24] Bates DW, Kuperman GJ, Rittenberg E, Teich JM, Fiskio J, et al (1999) A randomized trial of a computer-based intervention to reduce utilization of redundant laboratory tests. Am J Med 106(2): 144–150. 10230742 10.1016/s0002-9343(98)00410-0

[25] Nies J, Colombet I, Zapletal E, Gillaizeau F, Chevalier P, et al (2010) Effects of automated alerts on unnecessarily repeated serology tests in a cardiovascular surgery department: a time series analysis. BMC Health Serv Res 10: 70. 20298618 10.1186/1472-6963-10-70 PMC2848138

[26] Reed M, Huang J, Brand R, Graetz I, Neugebauer R, Fireman B, et al (2013) Implementation of an outpatient electronic health record and emergency department visits, hospitalizations, and office visits among patients with diabetes. JAMA 310: 1060–1065. 24026601 10.1001/jama.2013.276733 PMC4503235

[27] Hsiao CJ, Hing E (2012) Use and characteristics of electronic health record systems among office-based physician practies: United States, 2001–2012. NCHS data brief, no 111. Hyattsville, MD: National Center for Health Statistics. 23384787

[28] Grebely J, Matthews GV, Lloyd AR, Dore GJ (2013) Elimination of hepatitis C virus infection among people who inject drugs through treatment as prevention: Feasibility and future requirements. Clin Infect Dis. 57(7): 1014–20. 10.1093/cid/cit377 23728143

[29] Valenstein P, Leiken A, Lehmann C (1998) Test-ordering by multiple physicians increases unnecessary laboratory examinations. Arch Pathol Lab Med 112: 238–241. 3345123

[30] Kerr T, Wood E, Grafstein E, Ishida T, Shannon K, et al (2005) High rates of primary care and emergency department use among injection drug users in Vancouver. J Public Health 27: 62–66. 10.1093/pubmed/fdh189 15564279

[31] Knowlton AR, Hoover DR, Chung SE, Celentano DD, Vlahov D, Latkin CA (2011) Access to medical care and service utilization among injection drug users with HIV/AIDS. Drug Alcohol Depend 64: 55–62. 10.1016/s0376-8716(00)00228-3 11470341

[32] Chander G, Himelhoch S, Moore RD (2006) Substance abuse and psychiatric disorders in HIV-positive patients: epidemiology and impact on antiretroviral therapy. Drugs 66: 769–789. 16706551 10.2165/00003495-200666060-00004

[33] Krusi A, Wood E, Montaner J, Kerr T (2010) Social and structural determinants of HAART access and adherence among injection drug users. Int J Drug Policy 21: 4–9. 19747811 10.1016/j.drugpo.2009.08.003

[34] Yaphe S, Bozinoff N, Kyle R, Shivkumar S, Pai NP, Klein M (2012) Incidence of acute hepatitis C virus infection among men who have sex with men with and without HIV infection: a systematic review. Sex Transm Infect 88: 558–564. 22859499 10.1136/sextrans-2012-050566

[35] Lambers FA, Prins M, Davidovich U, Stolte IG (2014) High awareness of hepatitis C virus (HCV) but limited knowledge of HCV complications among HIV-positive and HIV-negative men who have sex with men. AIDS Care 26: 416–424. 24024525 10.1080/09540121.2013.832721

[36] Berwick DM, Hackbarth AD (2012) Eliminating waste in US health care. JAMA 307: 1513–1516. 22419800 10.1001/jama.2012.362

[37] McGregor MJ, Martin D (2012) Testing 1, 2, 3: is overtesting undermining patient and system health? Can Fam Physician 58: 1191–1193. 23152453 PMC3498009

[38] Yehia BR, Kangovi S, Frank I (2013) Patients in transition: avoiding detours on the road to HIV treatment success. AIDS 27: 1529–1533. 23435297 10.1097/QAD.0b013e328360104e

[39] Yehia BR, Schranz AJ, Momplaisir F, Keller SC, Gross R, Frank I, et al.. (2013) Outcomes of HIV-Infected Patients Receiving Care at Multiple Clinics. AIDS Behav Epub ahead of press Sept 28, 2013. 10.1007/s10461-013-0625-7 PMC3969411 24077931

[40] Lefevre F, Reifler D, Lee P, Sbenghe M, Nwadiaro N, Verma S, et al. (199) Screening for undetected mental disorders in high utilizers of primary care services. J Gen Intern Med 14: 425–431. 10417600 10.1046/j.1525-1497.1999.07238.x PMC1496609

[41] Neal RD, Heywood PL, Morley S (2000) Frequent attenders’ consulting patterns with general practitioners. Br J Gen Pract 50: 972–976. 11224969 PMC1313884

[42] Scaife B, Gill P, Heywood P, Neal R (2000) Socio-economic characteristics of adult frequent attenders in general practice: secondary analysis of data. Fam Pract 17: 298–304. 10934176 10.1093/fampra/17.4.298

[43] Liu P, Shi ZX, Zhang YC, Xu ZC, Shu HS, Zhang XY (1997) A prospective study of a serum-pooling strategy in screening blood donors for antibody to hepatitis C virus. Transfusion 37: 732–736. 9225938 10.1046/j.1537-2995.1997.37797369450.x

[44] Taylor LE, DeLong AK, Maynard MA, Chapman S, Gholam P, Blackard JT, et al (2011) Acute hepatitis C virus in an HIV clinic: a screening strategy, risk factors, and perception of risk. AIDS Patient Care STDS 25: 571–577. 21859307 10.1089/apc.2011.0106 PMC3183653

