# Supplementary material for: Hepatitis C Virus Testing in Adults Living with HIV: A Need for Improved Screening Efforts
Source: PLoS One. 2014 Jul 17;9(7):e102766. doi: 10.1371/journal.pone.0102766 (PMC4102540; doi:10.1371/journal.pone.0102766)
Supplement: Appendix S1 — Proportion of HCV Antibody Negative Patients with Repeat HCV Testing and Factors Associated with Repeat HCV Testing. (DOC) [file pone.0102766.s001.doc]

**Appendix Table1.** Proportion of HCV Antibody Negative Patients with Repeat HCV Testing and Factors Associated with Repeat HCV Testing.

| **Characteristics** | **Repeat HCV Testing**  **N=2,447 (%)** | **Adjusted Odds Ratio (95% CI)** |
| --- | --- | --- |
| **Age (years)**  18-29  30-39  40-49  ≥ 50 | 704 (42.08)  733 (43.17)  719 (43.13)  291 (41.45) | 1.00 (reference)  0.94 (0.79 – 1.11)  0.91 (0.76 – 1.09)  0.83 (0.65 – 1.05) |
| **Sex**  Male  Female | 1,831 (41.89)  616 (45.00) | 1.00 (reference)  1.04 (0.87 – 1.25) |
| **Race/Ethnicity**  White  Black  Hispanic  Other/Unknown | 596 (40.60)  1,164 (42.39)  610 (47.14)  77 (33.19) | 1.00 (reference)  1.06 (0.88 – 1.28)  0.94 (0.76 – 1.16)  0.89 (0.60 – 1.33) |
| **HIV Risk Factor**  MSM  HET  IDU  Other/Unknown | 1,277 (42.88)  957 (43.38)  99 (44.80)  114 (34.03) | 1.00 (reference)  1.03 (0.86 – 1.23)  1.54 (1.04 – 2.29)  0.93 (0.68 – 1.28) |
| **Insurance**  Private  Medicaid  Medicare  Ryan White/Uninsured  Other/Unknown | 331 (36.10)  647 (45.28)  192 (49.36)  1,052 (43.35)  225 (38.93) | 1.00 (reference)  1.17 (0.91 – 1.52)  1.54 (1.10 – 2.17)  1.26 (0.97 – 1.63)  0.91 (0.65 – 1.27) |
| **CD4 Cell Count (cell/mm^3^ )**  ≤ 350  351-500  > 500  Missing | 1,398 (42.88)  442 (41.70)  486 (42.59)  121 (43.37) | 1.00 (reference)  1.12 (0.94 – 1.33)  1.18 (0.99 – 1.41)  0.58 (0.40 – 0.85) |
| **Number of Outpatient HIV Visits***  ≤ 14  > 14 | 1,036 (30.22)  1,411 (61.03) | 1.00 (reference)  1.03 (1.02 – 1.04) |
| **Number of Inpatient Visits ***  0  ≥ 1 | 1,547 (38.30)  900 (52.91) | 1.00 (reference)  1.14 (1.08 – 1.20) |
| **Number of Emergency Department Visits ***  0  ≥ 1 | 1,380 (37.82)  1,067 (51.03) | 1.00 (reference)  1.00 (0.97 – 1.02) |
| **Observation Time (years)**  1  2  3  4  5  6 | 245 (10.48)  483 (24.03)  528 (35.53)  495 (40.57)  438 (45.25)  258 (45.42) | 1.00 (reference)  2.78 (2.24 – 3.45)  5.32 (4.19 –6.76)  6.76 (5.18 – 8.83)  10.78 (7.92 – 14.67)  12.30 (8.34 – 18.14) |

**Abbreviations:** CI, confidence interval; HET, heterosexual transmission; IDU, injection drug use; MSM, men who have sex with men.

* Number of outpatient HIV visits, inpatient visits, and emergency department visits were treated as continuous variables in multivariable logistic regression (column 2). These variables were dichotomized to facilitate calculation of proportions (column 1). The mean value divided the number of outpatient HIV visits during the observation period into two groups; whereas the number of inpatient and emergency department visits during the observation period differentiated between 0 and 1 or more visits.
